# Transcriptome Analysis of the Response of Mature *Helicobacter pylori* Biofilm to Different Doses of *Lactobacillus salivarius* LN12 with Amoxicillin and Clarithromycin

**DOI:** 10.3390/antibiotics11020262

**Published:** 2022-02-17

**Authors:** Fang Jin, Hong Yang

**Affiliations:** State Key Laboratory of Microbial Metabolism, School of Life Sciences and Biotechnology, Shanghai Jiao Tong University, Shanghai 201100, China; jinfang@sjtu.edu.cn

**Keywords:** *Lactobacillus salivarius*, *Helicobacter pylori*, biofilm, transcriptome, dose

## Abstract

*Helicobacter pylori* is a gastrointestinal pathogen with a high infection rate. Probiotics are clinically used as an adjuvant to improve the cure rate and reduce the side effects of antibiotic treatment for *H. pylori*. This study is the first to explore the effects of a cell-free supernatant of high- or low-dose *Lactobacillus salivarius* LN12 combined with amoxicillin (AMX) and clarithromycin (CLR) on *H. pylori* 3192 biofilms in terms of the biofilm biomass, survival rates, biofilm structure, and transcriptome. The results showed that the combination of the CFS of high-dose LN12 with AMX and CLR had stronger effects on the biofilm biomass, survival rate, and structure of *H. pylori* 3192 biofilms. *H. pylori* 3192 biofilms may increase the expression of NADH-related genes and downregulate flagellar assembly and quorum sensing-related receptor genes to deal with the stronger stress effects of high-dose LN12 with AMX and CLR. In conclusion, the biofilm biomass, survival rate, structure, and transcriptome results showed that the combination of LN12 CFS with AMX and CLR had dose effects. We recommend that compared with low doses, high doses of *L. salivarus* LN12 combined with AMX and CLR may be more effective for *H. pylori* biofilm than low doses.

## 1. Introduction

As microaerobic Gram-negative bacteria, *Helicobacter pylori* (*H. pylori*) can colonize the human stomach through the oral cavity and is closely linked to chronic gastritis, peptic ulcers, gastric mucosa-associated lymphoid tissue lymphoma, and other gastric disorders [1,2]. The global infection rate of *H. pylori* is more than 50% and affected by geographical and economic conditions, the infection rate in developing countries is higher than that in developed countries, and *H. pylori* was listed as a group I carcinogen by the World Health Organization [3]. The spirochete shape and flagellum structure of *H. pylori* help it move in the harsh low-pH environment of the stomach, and a variety of adhesion proteins and urease secreted by *H. pylori* facilitate its effective colonization. Two important virulence factors, cytotoxin-associated protein (CagA) and vacuolar cytotoxin (VacA), cause inflammation of gastric epidermal cells [4]. *H. pylori* can form biofilm structures in vivo and in vitro, which are composed of protein, mannan, LPS-related structures, extracellular DNA, proteins, and outer membrane vesicles. Biofilms can enhance resistance to antibiotics, improve the persistence and survival rate in the host, and reduce the success rate of treatment [5]. At present, the regulatory process of *H. pylori* biofilm formation has not been fully explained, and in particular, transcriptome research on *H. pylori* biofilms remains limited [6,7,8,9].

Antibiotics are the first-line therapy for the clinical treatment of *H. pylori*, and triple therapy, consisting of two antibiotics and a proton pump inhibitor, is the most widely used [2]. Amoxicillin (AMX) and clarithromycin (CLR) are the most commonly used antibiotics. However, antibiotic resistance is the main challenge in the treatment of *H. pylori*, as the use of antibiotics leads to the emergence of multidrug-resistant strains, diarrhea, vomiting, other adverse reactions, and changes in the gastrointestinal flora [2,10]. Some natural products have been reported to have therapeutic effects on *H. pylori,* such as extracts from *Myristica fragrans*, *Hibiscus rosa sinensis* flower, *Agrimonia eupatoria*, and *Fragaria vesca*, as have some special nanoparticles (fullerenol nanoparticles, special chitosan/poly acrylic acid particles co-loaded with superparamagnetic iron oxide nanoparticles and amoxicillin, chitosan-based nanoparticles) and *N*-acetylcysteine (NAC) (when in combination with clarithromycin and lansoprazole) [5,10,11,12,13,14,15,16,17]. Probiotic cell-free supernatant (CFS) can interfere with the growth of *H. pylori*, destroy *H. pylori* biofilms, hinder the adhesion of *H. pylori* in the stomach, and inhibit the immune response induced by *H. pylori* [18,19]. Compared with natural products, probiotics supplemented with antibiotics can not only improve the cure rate of *H. pylori* but also reduce adverse reactions to antibiotic treatment. However, the type, dose, interval, and treatment time of probiotics can affect the success rate of *H. pylori* treatment [20,21,22]. At present, there is no unified standard for the dose of clinical probiotics; 10^8^, 10^9^, and 10^10^ CFU are used, and no research has clearly shown that the higher the dose of probiotics in the treatment of *H. pylori* is, the better the effects will be [21].

At present, no study has explored the effects of the CFS of different doses of probiotics combined with AMX and CLR on *H. pylori* biofilms, especially on the transcriptome. Our previous studies showed that *Lactobacillus salivarius* (*L. salivarus*) LN12 CFS could enhance the destructive effects of AMX and CLR on *H. pylori* 3192 biofilm in vitro [23]. This study was the first to explore the effects of CFS of different doses of *L. salivarus* LN12 combined with AMX and CLR on *H. pylori* 3192 biofilm biomass, survival rate, and structure, and especially on the transcriptome.

## 2. Results

### 2.1. Effects of CFSs of Different Doses of L. salivarus LN12 in Combination with AMX and CLR on the Biomass of H. pylori Biofilms

**Figure 1:**
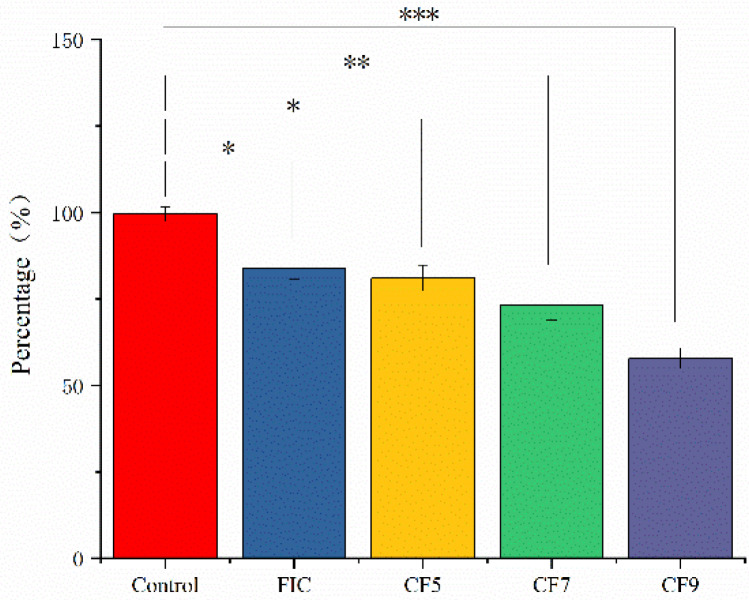
Effects of different treatments on *H. pylori* 3192 biofilm biomass. Four-day mature *H. pylori* biofilm was treated with different treatments, and the biomass of the *H. pylori* biofilm was assessed by 1% crystal violet staining. Control group: BB2, FIC group: 1 × FIC of AMX and CLR, CF5 group: 6.25% CFS of 10^5^ CFU/mL *L. salivarus* LN12 in combination with 1 × FIC of AMX and CLR, CF7 group: 6.25% CFS of 10^7^ CFU/mL LN12 in combination with 1 × FIC of AMX and CLR, CF9 group: 6.25% CFS of 10^9^ CFU/mL *L. salivarus* LN12 in combination with 1 × FIC of AMX and CLR. AMX: Amoxicillin, CLR: Clarithromycin. ∗: *p* ≤ 0.05, ∗∗: *p* ≤ 0.01, and ∗∗∗: *p* ≤ 0.001.

After washing twice with PBS, 4-day mature *H. pylori* 3192 biofilms on coverslips were subjected to different treatments for 12 h, and the biomass was quantified with the crystal violet staining method. The different treatments were as follows: AMX and CLR at FIC concentrations alone (FIC group) or combined with 6.25% CFS of 10^5^ CFU/mL (CF5 group), 10^7^ CFU/mL (CF7 group), or 10^9^ CFU/mL (CF9 group) *L. salivarus* LN12. BB2 was used as a negative control. Figure 1 showed that compared to the control group, FIC, CF5, CF7, and CF9 all significantly decreased the biomass (*p* ≤ 0.05), and CFS of different doses of *L. salivarus* LN12 could enhance the destructive effects of FIC on biofilms. With the increasing doses of *L. salivarus* LN12, the effects of CFS combined with AMX and CLR on biofilms were more significant.

### 2.2. Effects of CFSs of Different Doses of L. salivarus LN12 in Combination with AMX and CLR on the Viability of H. pylori Biofilms

**Figure 2:**
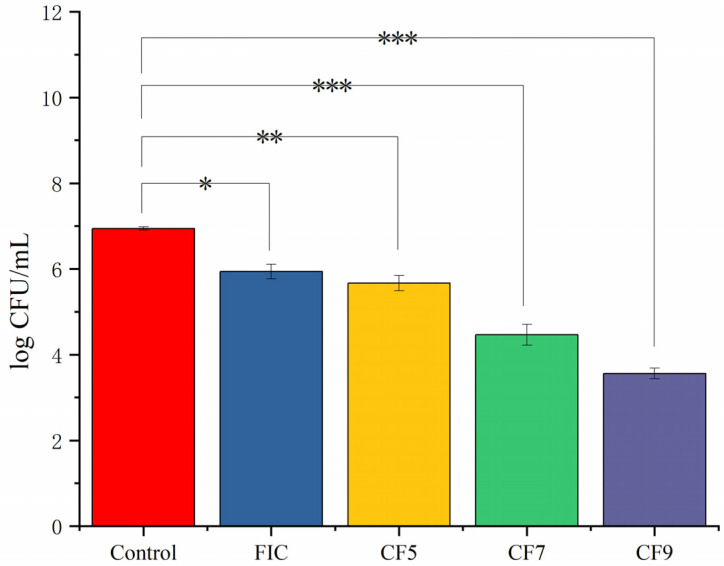
Effects of different treatments on the survival rate of *H. pylori* 3192 biofilm. Four-day mature *H. pylori* biofilm was treated with different treatments, and the survival rate was counted on a blood agar plate after 72 h. Control group: BB2, FIC group: 1 × FIC of AMX and CLR, CF5 group: 6.25% CFS of 10^5^ CFU/mL *L. salivarus* LN12 in combination with 1 × FIC of AMX and CLR, CF7 group: 6.25% CFS of 10^7^ CFU/mL *L. salivarus* LN12 in combination with 1 × FIC of AMX and CLR, CF9 group: 6.25% CFS of 10^9^ CFU/mL *L. salivarus* LN12 in combination with 1 × FIC of AMX and CLR. AMX: Amoxicillin, CLR: Clarithromycin. ∗: *p* ≤ 0.05, ∗∗: *p* ≤ 0.01, and ∗∗∗: *p* ≤ 0.001.

CFSs of different doses of *L. salivarus* LN12 were combined with AMX and CLR according to the above methods. The survival rate of *H. pylori* 3192 biofilms on coverslips under different treatments was evaluated by counting CFUs on blood plates. Figure 2 showed that the combination of CFSs of different doses of *L. salivarus* LN12 with AMX and CLR decreased the biofilm bacterial survival rate (*p* ≤ 0.05). CFS enhanced the destructive effects of FIC on the biofilm survival rate, and these effects became stronger with increasing doses of *L. salivarus* LN12.

### 2.3. Effects of CFSs of Different Doses of L. salivarus LN12 in Combination with AMX and CLR on the Structure of H. pylori Biofilms

**Figure 3:**
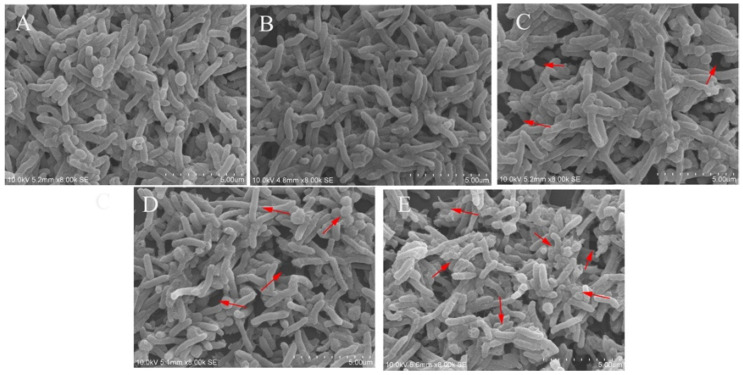
Effects of different treatments on a 4-day mature *H. pylori* 3192 biofilm under SEM. (**A**) (Control group): BB2, (**B**) (FIC group): 1 × FIC of AMX and CLR, (**C**) (CF5 group): 6.25% CFS of 10^5^ CFU/mL *L. salivarus* LN12 in combination with 1 × FIC of AMX and CLR, (**D**) (CF7 group): 6.25% CFS of 10^7^ CFU/mL *L. salivarus* LN12 in combination with 1 × FIC of AMX and CLR, (**E**) (CF9 group): 6.25% CFS of 10^9^ CFU/mL *L. salivarus* LN12 in combination with 1 × FIC of AMX and CLR. Arrows indicated the pores in biofilm and bacterial fragmentation. The magnification is 8000×, the scale bar is 5.00 μm. AMX: Amoxicillin, CLR: Clarithromycin.

The effects of the different treatments on *H. pylori* 3192 biofilms were observed by scanning electron microscopy (SEM). Figure 3 showed that the biofilm in the control group (A) was dense with a small number of holes, and the bacteria were intertwined and arranged in an orderly way. The bacteria were mainly spiral rod-shaped, accompanied by a small number of spherical shapes, which is consistent with existing literature reports [24]. Compared with the control group (A), the biofilm structure under different treatments (B, C, D, E) was damaged, and the combination of *L. salivarus* LN12 CFS with AMX and CLR enhanced the damage to the biofilm caused by the antibiotics. When combined with AMX and CLR, as the dose of *L. salivarus* LN12 increased, the proportion of the rod-shaped morphology of bacteria in the biofilm was significantly decreased while the proportion of spherical bacteria increased, more holes appeared, and more fragmentation was observed (especially in the area marked with an arrow in Figure 3).

**Figure 4:**
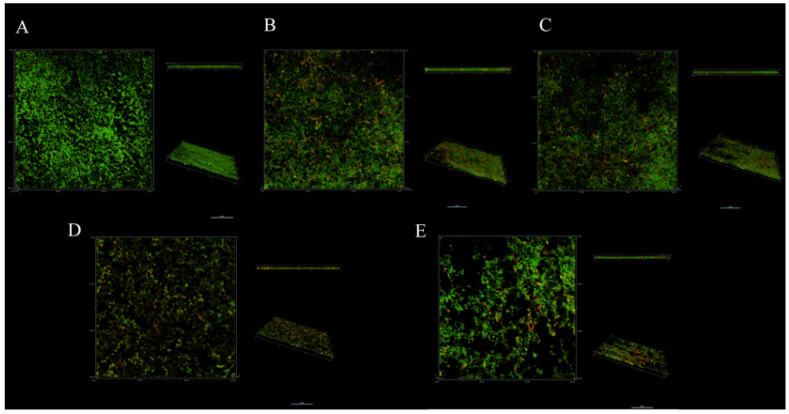
Effects of different treatments on *H. pylori* biofilm by CLSM. (**A**) (Control group): BB2, (**B**) (FIC group): 1× FIC of AMX and CLR, (**C**) (CF5 group): 6.25% CFS of 10^5^ CFU/mL *L. salivarus* LN12 in combination with 1 × FIC of AMX and CLR, (**D**) (CF7 group): 6.25% CFS of 10^7^ CFU/mL *L. salivarus* LN12 in combination with 1 × FIC of AMX and CLR, (**E**) (CF9 group): 6.25% CFS of 10^9^ CFU/mL *L. salivarus* LN12 in combination with 1 × FIC of AMX and CLR. The scale bar is 10 μm. Every group contains three views: the main view (observed horizontally along the x-y axis), the side view (observed horizontally along the x-z axis), and the view at a certain angle. AMX: Amoxicillin, CLR: Clarithromycin.

SYTO9 and PI can specifically combine with living and dead bacteria in biofilms. Under confocal laser scanning microscopy (CLSM), living bacteria and dead bacteria are green and red, respectively, under excitation at 488 and 560 nm, enabling visualization of the distribution of bacteria in biofilms (Figure 4). Dense green fluorescent clusters were observed in three views of the control group, and almost no red fluorescence was found in the side view, indicating that the untreated biofilm had a compact structure, and the proportion of dead cells under CLMS was almost zero (Figure 4A). After different treatments, the biofilm structure changed to varying degrees (Figure 4B–E). It can be seen from three angles that the biofilm was dispersed to varying degrees. The combination of *L. salivarus* LN12 CFS and AMX and CLR can enhance the destructive effects of antibiotics on the biofilm (Figure 4C–E), and the destructive effects increased as the dose of *L. salivarus* LN12 increased: the biofilm structure became looser, the distribution of green cells was smaller, and the proportion of dead bacteria (red) was larger, which is consistent with the SEM and biofilm viability results.

### 2.4. Transcriptome Analysis

**Figure 5:**
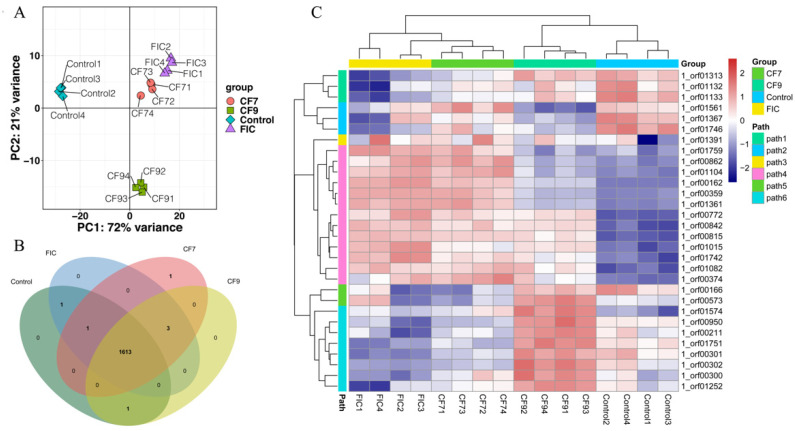
*H. pylori* 3192 biofilm transcriptome in different treatment groups. Principal component analysis (PCA) (**A**), Venn diagram (**B**), and heat map (**C**) showed the common and differently expressed genes in different treatment groups. Control group: BB2, FIC group: 1 × FIC of AMX and CLR, CF7 group: 6.25% CFS of 10^7^ CFU/mL *L. salivarus* LN12 in combination with 1 × FIC of AMX and CLR, CF9 group: 6.25% CFS of 10^9^ CFU/mL *L. salivarus* LN12 in combination with 1 × FIC of AMX and CLR. Four independent biological samples were used in every group.

To explore the effects of CFSs of different doses of *L. salivarus* LN12 combined with AMX and CLR on the *H. pylori* 3192 transcriptome, the cell-free supernatants (CFSs) of 10^7^ and 10^9^ CFU/mL *L. salivarus* LN12 were diluted to concentrations of 6.25%, then combined with FIC of AMX and CLR, and named the low-dose group (CF7) and high-dose group (CF9). AMX and CLR at the FIC values were used as the FIC group and BB2 as the blank control group (control). Then, 4-day *H.* pylori 3192 biofilms were treated with FIC, CF7, CF9, and BB2 for 12 h, and the changes in the biofilm transcriptome were observed. The RNA of all samples met the requirements of RNA-Seq (Supplementary Table S1). The density distribution analysis of the transcriptome expression showed that the structure of all groups was basically the same, with most genes showing medium expression (Supplementary Figure S1). The default screening conditions of differentially expressed genes (DEGs) were FDR ≤ 0.05 and |log2FC| ≥ 1, and the correction method for multiple tests was BH (fdr correction with Ben-jamini/Hochberg). The volcano plot (Supplementary Figure S2) showed 694 DEGs, 345 downregulated genes, and 349 upregulated genes in the FIC group compared with the control group; 584 DEGs, 286 downregulated genes, and 298 upregulated genes in the CF7 group compared with the control group; 625 DEGs, 315 downregulated genes, and 310 upregulated genes in the CF9 group compared with the control group; 46 DEGs, 11 downregulated genes, and 35 upregulated genes in the CF7 group compared with the FIC group; 400 DEGs, 190 downregulated genes, and 210 upregulated genes in the CF9 group compared with the FIC group; and 225 DEGs, 121 downregulated genes, and 104 upregulated genes in the CF7 group compared with the CF9 group. PCA showed that the CF7 group was correlated with the FIC group but presented a different state from the control and CF9 groups (Figure 5). The Venn diagram (B) and heatmap (C) showed that the four groups were in different states but were related to each other. FIC, CF7, and CF9 were different from the control group, but the states of CF7 and FIC were more similar, and the differences between CF9 and FIC were greater.

#### 2.4.1. GO Enrichment Analysis

**Figure 6:**
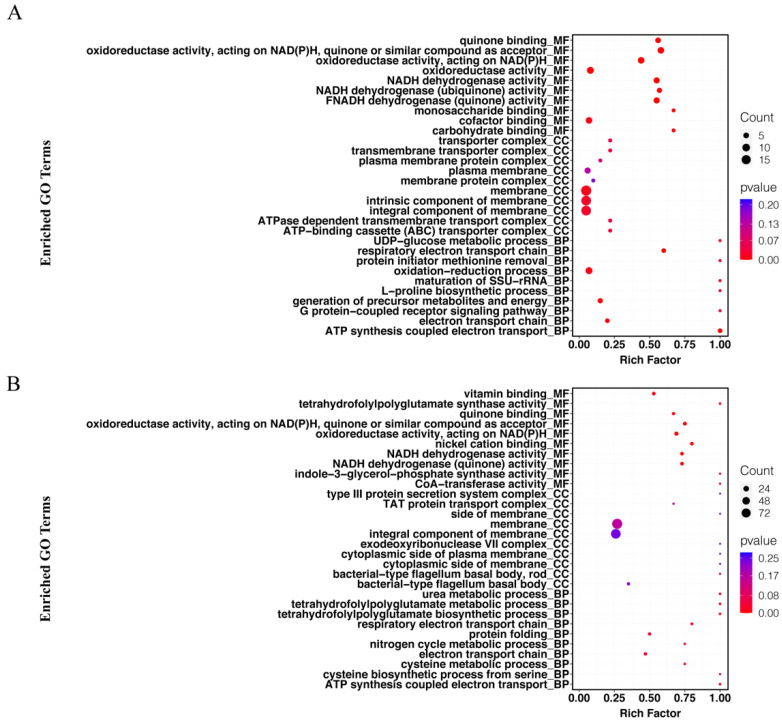
Analysis of GO enrichment for *H. pylori* 3192 biofilm under different treatments. Top 10 GO terms in the cellular component (CC), molecular component (MF), and biological process (BP) are shown. Rich factor refers to the ratio of DEGs enriched in the pathway to the number of genes annotated. The greater the value is, the higher the enrichment degrees. The size of the solid circle represents the number of DEGs enriched in this pathway, and the color represents the significance of enrichment. FIC group: 1 × FIC of AMX and CLR, CF7 group: 6.25% CFS of 10^7^ CFU/mL *L. salivarus* LN12 in combination with 1 × FIC of AMX and CLR, CF9 group: 6.25% CFS of 10^9^ CFU/mL *L. salivarus* LN12 in combination with 1 × FIC of AMX and CLR. (**A**) CF7 group vs. FIC group, (**B**) CF9 group vs. FIC group.

**Figure 7:**
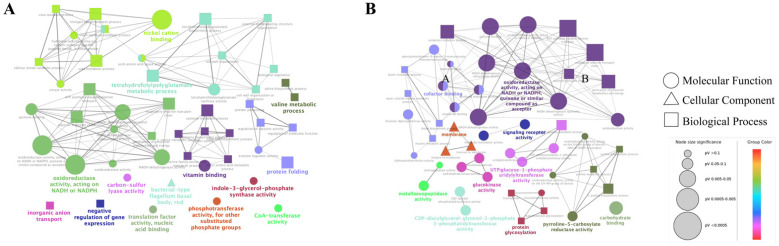
ClueGO analysis for GO terms (*p* < 0.1) in *H. pylori* 3192 biofilm under different treatments. Each node in the graph represents a GO term, the connection between nodes reflects the correlation between terms, and the color of the nodes reflects the enrichment and classification of the node. Significant GO terms are marked with color. FIC group: 1 × FIC of AMX and CLR, CF7 group: 6.25% CFS of 10^7^ CFU/mL *L. salivarus* LN12 in combination with 1 × FIC of AMX and CLR, CF9 group: 6.25% CFS of 10^9^ CFU/mL *L. salivarus* LN12 in combination with 1 × FIC of AMX and CLR. (**A**): CF7 group vs. FIC group, (**B**): CF9 group vs. FIC group.

We focused mainly on the effects of CFSs of different doses of *L. salivarus* LN12 combined with AMX and CLR on the *H. pylori* 3192 biofilm transcriptome. In the following section, the AMX- and CLR-treated group (FIC) was used as the control, and CFSs of high or low doses of *L. salivarus* LN12 combined with AMX and CLR were used as treatment groups (CF7 or CF9). GO functional annotation and enrichment analysis were carried out using the GO database. The top 10 GO terms with the lowest *p* values in cellular component (CC), molecular function (MF), and biological process (BP) were collected, and the first 5 GO terms were analyzed, as shown in Figure 6. For BP, compared to the FIC group, the most significant GO enrichment terms in the CF7 and CF9 groups were the respiratory electron transport chain and electron transport chain, and the largest numbers of DEGs in CF9 and CF7 were related to the electron transport chain and oxidation–reduction process, respectively. For CC, compared to the FIC group, the most significant GO enrichment terms in the CF7 and CF9 groups were the membrane and integral component of membrane, and CF9 had more DEGs. For MF, compared to the FIC group, the top five GO terms in CF7 and CF9 shared high similarity in oxidoreductase activity acting on NAD(P)H, NADH dehydrogenase activity, and quinone binding. The main difference between CF7 and CF9 was nickel cation binding, which was the third significant GO term in CF9. Furthermore, ClueGo was used to further cluster and visually analyze GO terms with *p* < 0.1 in CC, MF, and BP. Figure 7 shows that compared with the FIC group, the CF7 group obtained 11 final KappaScore groups and CF9 obtained 14. The most significant GO terms (color labeled) in each KappaScore group were also different in CF7 and CF9. Overall, compared with the FIC group, CF9 and CF7 shared some GO enrichment terms but also had distinct terms, and CF9 had higher metabolic vigor.

#### 2.4.2. KEGG Enrichment Analysis

**Figure 8:**
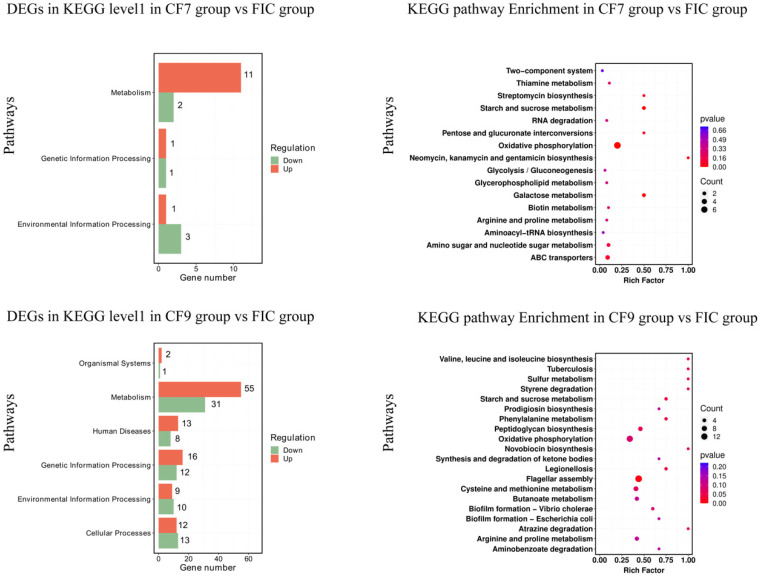
KEGG pathway and enrichment for *H. pylori* 3192 biofilm under different treatments. For the KEGG pathway database, biological metabolic pathways were divided into cellular processes, environmental information processing, genetic information processing, human diseases, metabolism, and biological systems in level 1. Rich factor refers to the ratio of DEGs enriched by the pathway to the number of genes annotated. The greater the factor is, the higher the enrichment degree is. For the enrichment analysis results, the size of the solid circle represents the number of DEGs enriched on this path, and the color represents the significance of enrichment. FIC group: 1 × FIC of AMX and CLR, CF7 group: 6.25% CFS of 10^7^ CFU/mL *L. salivarus* LN12 in combination with 1 × FIC of AMX and CLR, CF9 group: 6.25% CFS of 10^9^ CFU/mL *L. salivarus* LN12 in combination with 1 × FIC of AMX and CLR.

The distributions of the DEGs of CF7 and CF9 are shown in Figure 8. Compared with the FIC group, the DEGs in CF7 were related to metabolism (carbohydrate metabolism, energy metabolism, lipid metabolism, amino acid metabolism, metabolism of cofactors and vitamins, and biosynthesis of other secondary metabolites), genetic information processing (translation, folding, sorting, and degradation), and environmental information processing (membrane transport, signal transduction). In addition to the above pathways, DEGs were found to be related to metabolism (nucleotide metabolism, metabolism of cofactors and vitamins, metabolism of terpenoids and polyketides, biosynthesis of other secondary metabolites, and xenobiotic biodegradation and metabolism), organismal systems (immune system, environmental adaptation), human diseases (endocrine and metabolic diseases, infectious diseases: bacteria, drug resistance: antimicrobials), genetic information processing (transcription, replication, and repair), and cellular processes (flagellar assembly, biofilm formation, bacterial chemotaxis, quorum sensing) in CF9. Overall, compared with the FIC group, CF9 had more DEGs than CF7, and the number of KEGG pathways enriched in CF9 was greater. The significant pathway differences between the CF7 and CF9 groups were mainly reflected in metabolism, genetic information processing, and environmental information processing, especially in flagellar assembly, bacterial chemotaxis, quorum sensing, and antibiotic resistance for CF9.

Compared with the FIC group, the number of DEGs related to metabolism was the highest in CF7 and CF9. In total, 11 genes in the CF7 group were upregulated and 2 were downregulated while 55 genes in the CF9 group were upregulated and 31 were downregulated. The KEGG pathways shared by the CF7 and CF9 groups were glycolysis/gluconeogenesis, pentose and glucuronate interconversions, galactose metabolism, starch and sucrose metabolism, amino sugar and nucleotide sugar metabolism, oxidative phosphorylation, glycerophospholipid metabolism, arginine and proline metabolism, thiamine metabolism, biotin metabolism, streptomycin biosynthesis, neomycin, kanamycin, and gentamicin biosynthesis. CF9 also had DEGs related to the citrate cycle (TCA cycle), butanoate metabolism, carbon fixation pathways in prokaryotes, fatty acid biosynthesis, purine metabolism, and pyrimidine metabolism. The number of DEGs in oxidative phosphorylation in the CF7 and CF9 group was the largest, but the number in CF9 was greater than that in CF7, and the genes encoding *Helicobacter pylori* NADH-quinone oxidoreductase subunits M, J, L, NuoK, NuoH, NuoI, and NuoN were upregulated in both CF7 and CF9: 2.9-4.3-fold in CF7 and 5.7-8.3-fold in CF9. The genes encoding *Helicobacter pylori* NADH-quinone oxidoreductase subunits G and D and the fumarate reductase iron-sulfur subunit were upregulated 2.2-3.3-fold in CF9. The gene encoding *Helicobacter pylori* ATP F0F1 synthase subunit B was downregulated 0.4-fold in CF9. In starch and sucrose metabolism, the genes encoding UTP-glucose-1-phosphate uridylyltransferase GalU and glucokinase were upregulated 2.3-2.7-fold in CF7 and 3.2-4.4-fold in CF9, and the glucokinase gene was upregulated 3.6-fold in CF9. In genetic information processing, the KEGG pathways shared by CF7 and CF9 were aminoacyl-tRNA biosynthesis and RNA degradation. On this basis, CF9 contains 10 other pathways, including ribosome and protein export. The gene encoding *Helicobacter pylori* Asp-tRNA(Asn)/Glu-tRNA(Gln) amidotransferase subunit GatC was upregulated 2.7-fold in CF7; the genes encoding *Helicobacter pylori* Asp-tRNA(Asn)/Glu-tRNA(Gln) amidotransferase subunits GatC and GatB, phenylalanine--tRNA ligase subunit beta, and proline-tRNA ligase were upregulated 2.15-9.0-fold in CF9; and the *argS* gene was downregulated 0.44-fold in CF9.

For environmental information processing, the CF7 and CF9 group shared DEGs in ABC transporters and the two-component system. For ABC transporters, the genes encoding LPS export ABC transporter ATP-binding protein, cysteine ABC transporter permease, and transporter substrate-binding domain-containing protein were downregulated 0.44-0.47-fold in CF7, and the genes encoding ABC transporter substrate-binding protein, ABC transporter ATP-binding protein, ATP-binding cassette domain-containing protein, and osmoprotection protein were downregulated 0.37-0.48-fold in CF9. In the 2-component system, the genes encoding *Helicobacter pylori* TIGR00366 family proteins were upregulated 2.2-fold in CF7 and CF9; the genes encoding RNA polymerase sigma factor fliA and czcA were upregulated 2.2-3.8-fold in CF9; and the genes encoding McpB, flagellin, and flagellar motor stator protein were downregulated 0.20-0.44-fold in CF9. Compared with the CF7 group, the same gene in the same KEGG pathway was upregulated by a higher multiple in CF9, and the number of pathways was greater in CF9.

Flagellar assembly, quorum sensing, bacterial chemotaxis, and antibiotic resistance are all related to *H. pylori* biofilms. Compared with the FIC group, DEGs in the above pathways were not found in CF7 but were found in CF9. In total, 14 DEGs were related to flagellar assembly: the genes encoding flagellar motor switch proteins fliY and fliM and the RNA polymerase sigma factor fliA were upregulated 2.6-3.8-fold while the genes encoding flagellar hook protein flgE, flagellar basal body rod protein flgC, and flagellin were downregulated 0.15-0.45-fold. In total, 4 DEGs were related to quorum sensing: the genes encoding anthranilate synthase, membrane protein insertase YidC, and S-ribosylhomocysteinase were upregulated 2.0-3.0-fold, and the gene encoding preprotein translocase subunit SecG was downregulated 0.44-fold. In total, 4 DEGs were related to bacterial chemotaxis: the genes encoding flagellar motor switch proteins fliY and fliM were upregulated 2.5-3.0-fold, and the genes encoding HAMP domain-containing protein, ABC transporter substrate-binding protein, flagellar motor protein MotB, and flagellar motor stator protein were downregulated 0.38-0.44-fold. In total, 7 DEGs were related to biofilm formation: the genes encoding RNA polymerase sigma factor fliA, S-ribosylhomocysteinase, and anthranilate synthase were upregulated 2.0-3.8-fold, and the gene encoding serine O-acetyltransferase was downregulated 0.44-fold. In total, 5 DEGs were related to antimicrobial drug resistance: the genes encoding copper-translocating P-type ATPase CopA and aminotransferase class I/II-fold pyridoxal phosphate-dependent enzyme were upregulated 2.2-2.8-fold while the genes encoding penicillin-binding protein (pbp-1a), D-alanine ligase, and *murG* were downregulated 0.22-0.41-fold. Overall, the CF9 group was more active than the CF7 group in *H. pylori* biofilms, especially in flagellar assembly, bacterial chemotaxis, quorum sensing, and antibiotic resistance.

The top KEGG pathways with *p* value < 0.1 are shown in Supplementary Table S2. Compared with the FIC group, the pathways shared by the CF7 and CF9 groups were oxidative phosphorylation and starch and sucrose metabolism. The different pathways included carbohydrate metabolism, biosynthesis of other secondary metabolites, membrane transport in CF7, and amino acid metabolism, energy metabolism, biosynthesis of other secondary metabolites, xenobiotic biodegradation and metabolism, cell motility, and cellular community-prokaryotes in CF9.

Compared with the FIC group, the top 20 genes with the most upregulated and downregulated levels in CF7 and CF9 are listed in Supplementary Table S3. The most upregulated gene in CF7 was 1_orf01846, at 4.26-fold, which was related to *Helicobacter pylori* NADH-quinone oxidoreductase subunit M, and other genes related to NADH-quinone oxidoreductase subunit genes were upregulated almost 3-fold. The most upregulated gene in CF9 was 1_orf00834, with a 12.10-fold increase. Other genes related to the NADH-quinone oxidoreductase subunit were upregulated almost 5.67-fold. Overall, the most upregulated genes in both the CF7 and CF9 groups were related to NADH-quinone oxidoreductase, and the results are in accordance with KEGG and GO enrichment, all of which showed that the oxidative phosphorylation pathway was active. The most downregulated gene in CF7 was 1_orf01871, at 0.63-fold, which was related to thiamine diphosphokinase. The most downregulated gene in CF9 was adenosylmethionine-8-amino-7-oxononanoate transaminase, which was downregulated 0.13-fold. Interestingly, some flagellum-related genes were significantly downregulated. The results are in accordance with KEGG enrichment, and all showed that pathways related to flagellar assembly were significant in the CF9 group.

#### 2.4.3. RT-qPCR Validation

**Figure 9:**
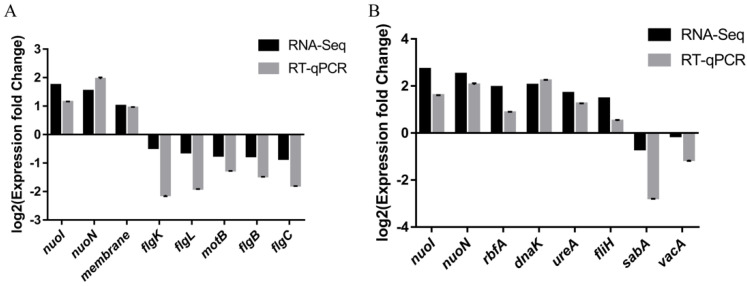
Validation of RNA-Seq by RT-qPCR on selected DEGs. FIC group: 1 × FIC of AMX and CLR, CF7 group: 6.25% CFS of 10^7^ CFU/mL *L. salivarus* LN12 in combination with 1 × FIC of AMX and CLR, CF9 group: 6.25% CFS of 10^9^ CFU/mL *L. salivarus* LN12 in combination with 1 × FIC of AMX and CLR. (**A**): CF7 vs. FIC, (**B**): CF9 vs. FIC.

To validate the RNA-Seq, eight DEGs for CF7 (*nuoI*, *nuoN*, *membrane*, *flgK*, *flgL*, *motB*, *flgB*, *flgC*) and eight DEGs *(nuoI*, *nuoN*, *rbfA*, *dnaK*, *ureA*, *fliH*, *sabA*, *vacA*) for CF9 were chosen for RT–qPCR. The FIC group was used as the control group, and the results are shown in Figure 9. The change trend of the above genes was consistent in RT–qPCR and RNA-Seq.

## 3. Discussion

Probiotics have been clinically proven to affect the treatment of *H. pylori*, and clinicians recommend the combination of probiotics and antibiotics, but there is no unified standard regarding the strains, dose, timing, and course of treatment of probiotics. A meta-analysis showed that *L. plantarum* and *P. acidilactici* were used at a dose of 1×10^9^ CFU for 10 days, *L. rhamnosus* GG at a dose of 1.2 × 10^10^ CFU for 14 days, and *L. reuteri* DSM17938 and *L. reuteri* ATCC PTA 6475 at a dose of 2 × 10^8^ CFU for 13 weeks [18]. Our previous research found that the combination of *L. salivarus* LN12 CFS with AMX and CLR had stronger destructive effects on *H. pylori* biofilms in vitro, but no study has explored whether the combination of CFSs of different doses of *L. salivarus* LN12 with AMX and CLR has different effects on *H. pylori* biofilms in vitro, especially on the transcriptome [23]. Therefore, we evaluated the effects of CFSs of high- and low-dose *L. salivarus* LN12 combined with AMX and CLR on *H. pylori* biofilms in terms of physiological indices and transcriptomes.

*H. pylori* biofilms are related to culture conditions, culture systems, and strains in vitro. Biofilms may effectively protect *H. pylori* from antibacterial agents and the immune system [5]. There are two main methods for forming *H. pylori* biofilms in vitro: biofilms can be formed at the gas–liquid interface in liquid culture or grown on nitrocellulose (NC) membranes attached to blood agar plates [19,23,25,26]. *H. pylori* biofilms were previously used to screen some natural products with anti*-H. pylori* effects. The evaluation indices were the biofilm biomass, the bacterial survival rate in biofilm, and the biofilm structure [27,28,29]. The results of our previous study showed that the MIC of 10^9^ CFU/mL *L. salivarus* LN12 CFS to *H. pylori* 3192 was 12.5%, and that CFS of 10^9^ CFU/mL *L. salivarus* LN12 can enhance the destructive effects of AMX and CLR on *H. pylori* 3192 biofilm. The effects of CFS alone were verified, and the results indicated that some acids and antibacterial proteins in LN12 CFS had a killing effect on *H. pylori* 3192 biofilm. CFS did not contain hydrogen peroxide or contained too low a concentration of hydrogen peroxide, and the metabolites in CFS that played a role were thermally stable; these factors may have all had effects [23]. For better observation, 1/2 MIC (6.25%) of *L. salivarus* LN12 CFS was combined with AMX and CLR. To compare the effects of different doses of *L. salivarus* LN12 CFS combined with AMX and CLR, 6.25% CFS of 10^5^ CFU/mL, 10^7^ CFU/mL, and 10^9^ CFU/mL *L. salivarus* LN12 were used with AMX and CLR, named the CF5, CF7, and CF9 groups, respectively [23]. The results showed that the higher the dose of *L. salivarus* LN12 combined with AMX and CLR, the more significant the destruction of the biofilm biomass, survival rate, and structure, which may suggest that although probiotics have therapeutic effects on *H. pylori*, a certain dose is necessary to exert a significant therapeutic effect, and the effect of a low dose may be nonsignificant.

At present, there are few studies on the transcriptome of *H. pylori* biofilms. Hathrougbi compared the transcriptome of *H. pylori* SS1 in planktonic and biofilm forms at the gas–liquid interface [6]. *H. pylori* biofilms are difficult to analyze by RNA-Seq because when extracting RNA after different treatments, too long a treatment time or errors when operating the technology can cause RNA degradation. RNA-Seq requires high purity and integrity of the RNA. This experiment found that RNA from *H. pylori* 3192 biofilms subjected to different treatments needs to be extracted immediately, and quick freezing by liquid nitrogen leads to RNA degradation. The *H. pylori* biofilm contains sugars, proteins, etc., and we ground and homogenized the sample before extracting the RNA, which greatly increased the total amount of RNA. In this study, the transcriptome of *H. pylori* biofilms formed at the gas–liquid interface and after being subjected to different treatments, it was studied for the first time to provide a reference method for the transcriptome of *H. pylori* biofilms [23].

Compared with the FIC group, KEGG and GO enrichment analysis showed that a large number of DEGs in CF9 and CF7 were related to the oxidative phosphorylation process. *H. pylori*, an obligate microaerophilic bacterium, contains two NADH-quinone oxidoreductases in the respiratory chain: proton-translating NADH-quinone oxidoreductase (NDH-1 or complex I) and NADH-quinone reductase (NDH-2). NDH-1 is located on the inner membrane of mitochondria and catalyzes the transfer of electrons from NADH to the quinone pool through a series of redox centers [30,31]. When organisms are under stress, oxidative phosphorylation, carbon assimilation, and the tricarboxylic acid cycle are involved in antitoxicity processes. It was found that by increasing the time of exposure to sodium fluoride (NAF), the damage to silkworm testes became more serious, indicating that NaF stress affected the NADH respiratory chain of the mitochondrial electron transport chain, increased the activity of related enzyme complexes, and significantly increased the content of intracellular ROS, resulting in an intracellular oxidative stress response [32]. NADH is the main cofactor required to reduce toxic compounds to low toxic compounds. The level of NADH/NAD+ is related to oxidative stress. The response of *Salmonella typhimurium* to vanillin in apple juice was studied by transcriptome analysis, and it was found that some NADH-related genes were upregulated (*nuoA*, *nuoB*, *nuoK*), which increased the oxidative stress response [33]. When *H. pylori* 3192 biofilms were treated with the CFS of high-dose *L. salivarus* LN12 combined with AMX and CLR, the oxidative phosphorylation of biofilms was more active, and the increase in the expression of *nuoK*, *nuoH*, *nuoI*, *nuoN*, and other subunit genes of NDH-1 in CF9 was significantly higher than that in CF7. It was speculated that CF9 could cause stronger damage to *H. pylori* biofilm cells, causing *H. pylori* 3192 to enter a stronger stress state with increased NADH-related gene expression, and a stronger oxidative stress response to help “detoxify”. Physiological indicators showed that CF9 had stronger effects on the biomass, survival rate, and structure of *H. pylori 3192* biofilms.

Compared with the FIC group, some DEGs occurred in flagellar assembly, quorum sensing, bacterial chemotaxis, biofilm formation, and antimicrobial drug resistance in the CF9 group but not in the CF7 group. The gene regulation process of *H. pylori* biofilms has not been fully explained [34]. Hathrougbi compared the biofilm and planktonic forms of SS1 through transcriptome analysis and found that the genes related to flagellum formation (e.g., *flgL*, *flgK*, *flgB*, *flgE*, and *fliK*) were significantly upregulated and might be related to biofilm formation, and ΔmotB and ΔflimA were significantly downregulated in biofilms [6]. We found that after treatment with the CFS of high-dose *L. salivarus* LN12 and AMX and CLR, *flgK*, *flgL*, *flgE*, *flgB*, and *motB* were downregulated in *H. pylori* 3192 biofilms. The strong destructive effects of CF9 on the biomass, survival rate, and structure of *H. pylori* 3192 biofilms may be related to the downregulation of these genes.

Bacteria can regulate biofilms through quorum sensing communication. *H. pylori* has only one known quorum sensing system, automatic inducer 2 (AI-2), synthesized by the LuxS gene. *H. pylori* senses AI-2 as negative chemotaxis through the chemical receptor TlpB, and chemorepulsion by AI-2 is the decisive factor regarding the spatial organization and diffusion of *H. pylori* biofilms [34,35]. Early colonization of *Arabidopsis* roots by *Bacillus subtilis* is mediated by the characteristic chemical receptors McpB and McpC and TlpC, and Δ*mcpb* can significantly reduce this early colonization [36]. We found that the *mcpb* gene was significantly downregulated after *H. pylori* 3192 biofilms were treated with CF9. We speculate that the stronger destructive effect of CF9 on *H. pylori* 3192 biofilms may be related to the downregulation of the *mcpb* gene.

Studies have shown that subinhibitory concentrations (sub-MICs) of antibiotics increase bacterial tolerance to antibiotics and the risk of drug resistance. The FIC concentrations of AMX and CLR used in this study were lower than MIC, which raised the question of why DEGs related to drug resistance did not appear in the CF7 group distribution [37,38]. When we explored the effects of CFSs of different doses of *L. salivarus* LN12 in combination with antibiotics, DEGs related to drug resistance did not appear in the CF7 group when the FIC group was used as the control but did appear in the CF7 group when the BB2 group was used as the control. Fourteen DEGs were validated by qPCR: *nuoI* and *nuoN* were related to NDH-1 activity; *flgK*, *flgL*, *flgB*, *flgC*, *motB*, and *fliH* were related to flagella; *rbfA* was related to translation and regulation; *dnaK* was related to heat shock; and *sabA* and *vacA* were related to virulence [39,40,41,42,43,44,45,46,47]. In our previous study, the *vacA* gene was downregulated significantly when *L. salivarus* LN12 CFS was combined with AMX and CLR, which was confirmed in this study [23].

Yang found that probiotics did not show a dose effect on *Clostridioides difficile* (CD) in vitro through transcriptome analysis. High-dose *Bifidobacterium breve* YH68 may have failed because it induced CD to produce more toxins and spores, and low-dose YH68 may have failed because it stimulated CD to exhibit stronger drug resistance [48]. In this study, physiological indices and transcriptome results both showed that the effects of high-dose *L. salivarus* LN12 CFS combined with antibiotics on *H. pylori* 3192 biofilm were greater than that of low-dose *L. salivarus* LN12 CFS. We suggest that high-dose *L. salivarus* LN12 should be used in the clinical adjuvant treatment of *H. pylori*, and the effects of low-dose *L. salivarus* LN12 are not obvious. We propose the concept of a “threshold”, where the effects of probiotic may be more obvious when the dose is greater than the “threshold”. We also need to recognize that the dose effects of *L. salivarus* LN12 may be related to strain specificity. To facilitate the performance of the experiment, our laboratory used CFS instead of the *L. salivarus* LN12 strain.

## 4. Materials and Methods

### 4.1. Strains and Culture Conditions

*H. pylori* clinical isolate 3192 was provided by Renji Hospital of Shanghai Jiaotong University (Shanghai, China), and *Lactobacillus salivarius* (LN12) was provided by Jiaxing Innocul-Probiotics Co., Ltd. (Jiaxing, China). *H. pylori* was cultured on Columbia blood agar plates containing 5% sterile defibrinated blood or in Brinell broth medium containing 10% Gibco fetal bovine serum (BB10) for proliferation. *H. pylori* biofilms were cultured in Brinell broth medium containing 2% fetal bovine serum (BB2), all at 37 °C in a microaerobic environment (MGC, Tokyo, Japan) for 48–72 h, and the speed of liquid culture was set to 100 rpm. With reference to Jin and Ji, modified MRS medium was used for *L. salivarus* LN12 at 37 °C for 24–48 h [19,23].

### 4.2. H. pylori Biofilm Culture

The method of culturing *H. pylori* biofilm was reported by Jin: after activation for 2 generations, *H. pylori* was cultured in a 12-well sterile cell culture plate with a 20 mm × 20 mm borosilicate coverslip (Matsunami Glass, Tokyo, Japan) and 2 mL of BB2 in each well, and the initial concentration of *H. pylori* 3192 in each well was no less than 10^7^ CFU/mL. The speed was set at 80 rpm, and *H. pylori* biofilm was formed at the gas–liquid interface at 37 °C in microaerobic culture for 4 days [23].

### 4.3. Preparation of the Combination of Lactobacillus Salivarius LN12 Cell-Free Supernatant (CFS) and Antibiotics

According to our previous study, the FIC concentrations of AMX and CLR for *H. pylori* 3192 were 2 × MIC_CLR_ and 0.125 × MIC_AMX_, MIC_CLR_ was 0.016 mg/L, MIC_AMX_ was 0.016 mg/L, and the MIC of *L. salivarus* LN12 (10^9^ CFU/mL) CFS on *H. pylori* 3192 was 12.5% [23]. When AMX and CLR were used in combination with *L. salivarus* LN12 CFS, the concentration was set to 1 × FIC and 1/2 × MIC of *L. salivarus* LN12 CFS (6.25%) [23]. To compare the effects of the CFS from different doses of *L. salivarus* LN12 with antibiotics, CFSs from different doses of *L. salivarus* LN12 were all diluted to 6.25% as follows: after activation for 2 generations, *L. salivarus* LN12 was cultured in mMRS to the logarithmic phase at 37 °C; diluted with BB2 to concentrations of 10^9^, 10^7^, and 10^5^ CFU/mL; and then centrifuged at 7600 rpm and 4 °C for 10 min. The CFS was filtered with a 0.22 μm sterile filter membrane and diluted to 6.25% with BB2, then finally combined with 1 × FIC of AMX and CLR. According to the dose of *L. salivarus* LN12, the groups were named as follows: CF9 group (6.25% CFS of 10^9^ CFU/mL *L. salivarus* LN12 in combination with 1 × FIC of AMX and CLR), CF7 group (6.25% CFS of 10^7^ CFU/mL *L. salivarus* LN12 in combination with 1 × FIC of AMX and CLR), CF5 group (6.25% CFS of 10^5^ CFU/mL *L. salivarus* LN12 in combination with 1 × FIC of AMX and CLR), and FIC group (2 × MIC_CLR_ and 0.125 × MIC_AMX_), BB2 was set as the control.

### 4.4. Effects of Different Treatments on the Viability of H. pylori Biofilms

The viability of *H. pylori* biofilms after different treatments was determined according to the CFU counting method on plates with reference to Jin: 4-day *H. pylori* 3192 biofilms on coverslips cultured in a 12-well cell culture plate were washed twice with PBS and separately placed in 2 mL of CF9, CF7, CF5, FIC of AMX and CLR, and BB2 as the control at 80 rpm and 12 h under microaerobic conditions. After washing twice with PBS, the biofilm was resuspended in 1 mL of PBS with a cell scraper. CFUs in the biofilm were counted on Columbia agar plates containing 5% sterile defibrinated sheep blood under microaerobic conditions after 72 h [23].

### 4.5. Effects of Different Treatments on the Biomass of H. pylori Biofilms

The *H. pylori* 3192 biofilms were subjected to different treatments as described above. After washing twice with PBS, the biofilm on the coverslips was stained with 1% crystal violet for 20 min and then resuspended in 400 μL of 95% ethanol. The absorbance at a wavelength of 595 nm was measured by a spectrophotometer [23].

### 4.6. Effects of Different Treatments on the Structure of H. pylori Biofilm

*H. pylori* 3192 biofilms on coverslips were observed under scanning electron microscopy (SEM) and confocal laser scanning microscopy (CLSM) according to Jin [23]. After being subjected to different treatments as described above, for SEM observation, after fixation overnight with 50% glutaraldehyde and dehydration with 50–100% gradient ethanol, the biofilm was dried at the critical point, then sprayed with gold (Leica, Wetzlar, Germany) and observed under SEM (S-4800, Hitachi, Tokyo, Japan). For CLSM, the biofilm was placed in a 1:1 mixture of SYTO 9 and propidium iodide (Invitrogen, Carlsbad, CA, USA) for 20 min in the dark and then observed under CLSM (Nikon, Tokyo, Japan) using an oil objective. The excitation wavelengths were 488 and 560 nm, and the *Z*-axis acquisition signal step length was 0.1 μm. Nikon Ni-E A1 HD25 special software was used to analyze the image [23].

### 4.7. Preparation of RNA-Seq Sequencing Library

First, 4-day *H. pylori* 3192 biofilms cultured in 12-well cell culture plates were washed twice with PBS and separately placed in 2 mL of CF9, CF7, FIC of AMX and CLR, or BB2 (used as a control) at 80 rpm and 37 °C for 12 h under microaerobic conditions. Four independent biological samples were used for every group. *H. pylori* 3192 biofilms on the plate wall were collected with a cell scraper, and the extraction method to obtain biofilm RNA was slightly adjusted on the basis of Wang. Homogenate grinding at low temperature was carried out before extraction [49]. RNA was extracted with a TransZol Up Plus RNA Kit (TransGen Biotech, Beijing, China), and RNA quality and quantity were evaluated using 1% agarose gel electrophoresis, Qubit 3.0 (Thermo Fisher Scientific, Bannockburn, MA, USA), Nanodrop One (Thermo Fisher Scientific, Bannockburn, MA, USA), and Agilent 4200 (Agilent Technologies, Waldbron, Germany). An ALFA-SEQ rRNA Depletion Kit was used to remove ribosomal RNA. The library was constructed using the NEB Next UltraTM Directional RNA Library Prep Kit for Illumina (New England Biolabs, Ipswich, MA, USA), qualified libraries were generated using the Illumina NovaSeq6000 platform, and 150 bp paired-end reads were generated.

### 4.8. Bioinformatics Analysis

Clean reads were obtained by quality control of raw reads with Trimmomatic (v.0.36), ribosomal sequences were removed by Bowtie2 software, and reads were mapped to the annotated reference *Helicobacter pylori* strain 3192 chromosome complete genome in NCBI (GenBank: CP086760). The sequencing quality was judged by RSeQC software and a homemade script, and the expression was counted by RSEM software. The expression level was measured in fragments per kilobase per million reads (FPKM). Differential gene analysis was performed on multiple samples using read counts, differentially expressed genes (DEGs) were analyzed with edgeR (v3.16.5) with default screening conditions of FDR ≤ 0.05 and |log2(fold change)| ≥ 1, and the correction method of multiple inspection was BH (fdr correction with Ben-jamini/Hochberg). Principal component analysis (PCA), Venn diagrams, and heat maps were used to analyze the relationships between group samples. Cluster profiler (v3.4.4) was used to analyze the functions of the DEGs to determine the roles of these genes in cells and the metabolic pathways involved. The annotation databases used were Gene Ontology (GO) and Kyoto Encyclopedia of Genes and Genomes (KEGG). GO terms with *p* value < 0.1 were selected and further analyzed with the ClueGO plugin in Cytoscape (3.9.0), and *Helicobacter pylori* 26,695 was used as a loading marker [48,50].

### 4.9. RT-qPCR Validation

Fourteen DEGs were selected to validate the RNA-Seq results with RT–qPCR, and RNA from the control group, FIC group, CF7 group, and CF9 group was reverse transcribed with the PrimeScriptTM RT Reagent Kit with gDNA Eraser No. RR047A (Perfect Real Time) (Takara, Kyoto, Japan). QPCR was performed using TaKaRa TB Green Premix Ex Taq™ II (Tli RNaseH Plus) (Code No. RR820A) (Takara, Kyoto, Japan) on a quantitative PCR apparatus (Analytikjena, Jena, Germany). The primers used are listed in Supplementary Table S4, and the results were analyzed using the 2^−^^∆∆CT^ method. The 16S rRNA gene was used as an internal reference.

### 4.10. Data Analysis and Availability

All experimental data were expressed as the mean ± standard deviation (*n* = 3), and statistical analysis was performed by one-way analysis of variance (ANOVA) and Tukey’s multiple comparison test using OriginPro Learning Edition (OriginLab, Northampton, MA, USA). A *p* value ≤ 0.05 was considered statistically significant.

## 5. Conclusions

In this study, *H. pylori* 3192 biofilm was treated with CFSs of high- and low-dose *L. salivarus* LN12 combined with AMX and CLR for the first time in vitro. The changes in biofilms were characterized by physiological indices and transcriptomes. Compared with that of low-dose *L. salivarus* LN12, the CFS of high-dose *L. salivarus* LN12 combined with AMX and CLR had more significant destructive effects on the *H. pylori* 3192 biofilm biomass, survival rate and biofilm structure. The transcriptome results showed that compared with the AMX and CLR treatment group, the largest number of DEGs for treatment with high- and low-dose *L. salivarus* LN12 CFSs combined with AMX and CLR were related to oxidative phosphorylation; more DEGs were related to oxidative phosphorylation for the high-dose *L. salivarus* LN12 CFS; and in particular, DEGs related to flagellar assembly, bacterial chemotaxis, quorum sensing, antibiotic resistance, and *H. pylori* biofilm formation appeared in the high-dose group. Compared with the low-dose *L. salivarus* LN12 combination, *H. pylori* 3192 may produce a stronger oxidative stress response by increasing NADH-related genes and downregulating flagellar assembly-related and quorum sensing-related receptor genes to cope with the stronger destructive effects on the biofilms of high-dose *L. salivarus* LN12 in combination with AMX and CLR. This study suggests that when *L. salivarus* LN12 is combined with AMX and CLR for the treatment of *H. pylori* biofilms, the therapeutic effects of high-dose probiotics may be better than those of low-dose probiotics, and the effects may be more obvious when the dosage of probiotics is higher than a certain “threshold”.

## Data Availability

Raw data are publicly available at Sequence Reads Archive with sample name SAMN23509605 (Control group), SAMN23509606 (FIC group), SAMN23509607 (CF7 group), SAMN23509608 (CF9 group).

## Supplementary Materials

The following supporting information can be downloaded at: https://www.mdpi.com/article/10.3390/antibiotics11020262/s1, Supplementary Figure S1: Sample expression pattern, Supplementary Figure S2: Volcano plot, Supplementary Table S1: RNA sample quality results, Supplementary Table S2: Top KEGG pathway (*p* < 0.1) in CF7 and CF9, Supplementary Table S3: The top 20 up- and downregulated genes in CF7 and CF9, Supplementary Table S4: Real-time PCR primers.
