# Supplementary figures and images for: Transcriptome Analysis of the Response of Mature Helicobacter pylori Biofilm to Different Doses of Lactobacillus salivarius LN12 with Amoxicillin and Clarithromycin
Source: Antibiotics (Basel). 2022 Feb 17;11(2):262. doi: 10.3390/antibiotics11020262 (PMC8868532; doi:10.3390/antibiotics11020262)

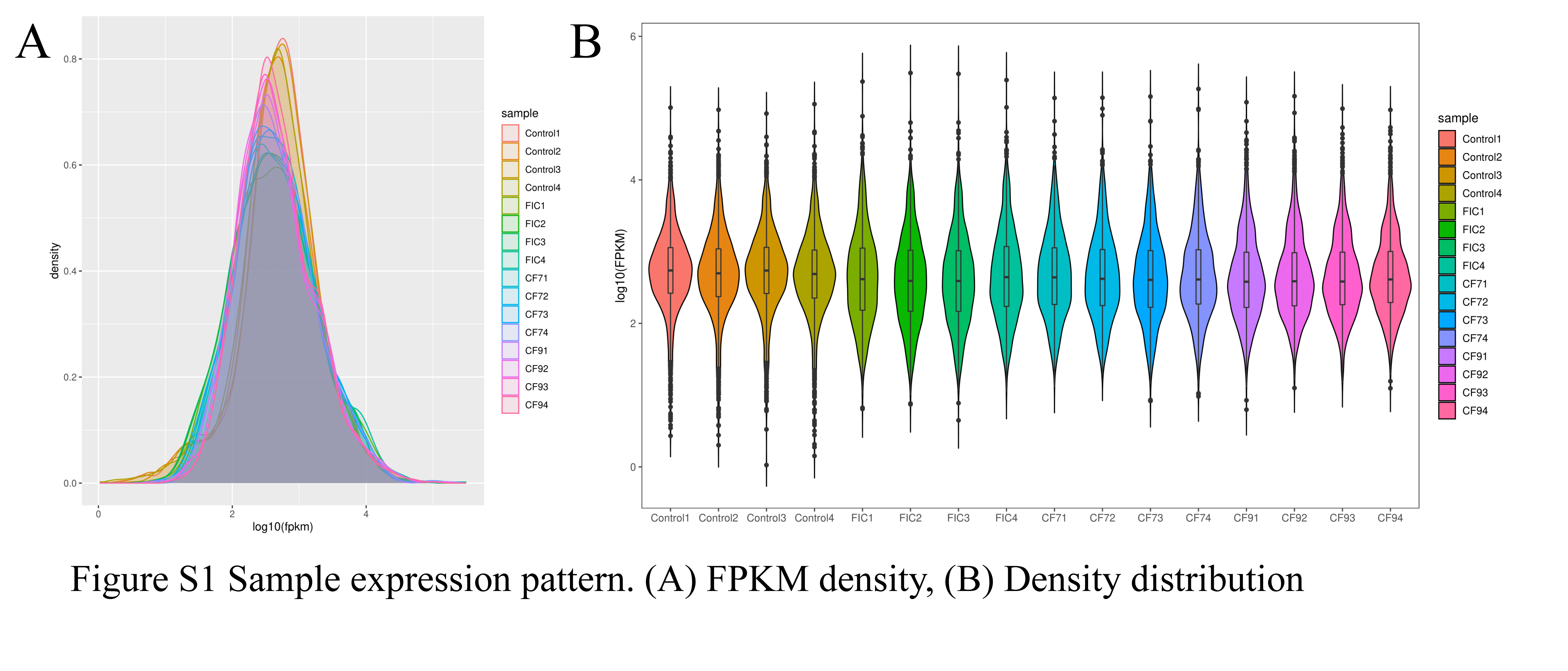

Supplement: Supplementary file 1 [file antibiotics-11-00262-s001.zip › Supplementary Files/Supplementary Figure S1.tif]

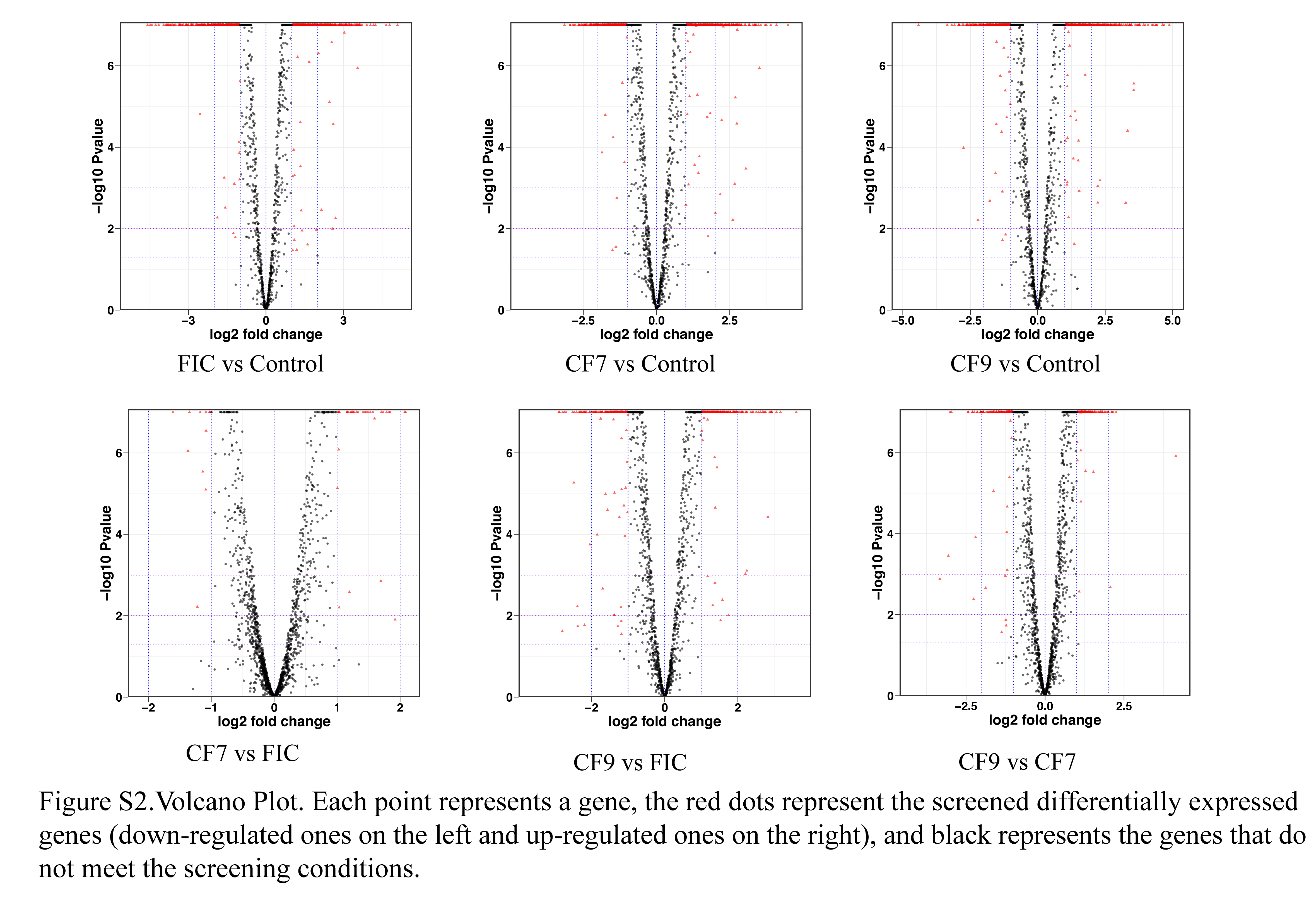

Supplement: Supplementary file 1 [file antibiotics-11-00262-s001.zip › Supplementary Files/Supplementary Figure S2.tif]
